# Effects of AgNPs on the Snail *Biomphalaria glabrata*: Survival, Reproduction and Silver Accumulation

**DOI:** 10.3390/toxics7010012

**Published:** 2019-03-01

**Authors:** Eduardo Cyrino Oliveira-Filho, Daphne Heloísa de Freitas Muniz, Esther Lima de Carvalho, Paolin Rocio Cáceres-Velez, Maria Luiza Fascineli, Ricardo Bentes Azevedo, Cesar Koppe Grisolia

**Affiliations:** 1 Embrapa Cerrados, Rodovia BR 020, Km 18, Planaltina, DF 73301-970, Brazil; daphne.muniz@embrapa.br; 2 Centro Universitário de Brasília (UniCEUB), Faculdade de Ciências da Educação e Saúde (FACES), Brasília, DF 70790-075, Brazil; esther.lima.21@gmail.com; 3 Instituto de Ciências Biológicas, Departamento de Genética e Morfologia, Universidade de Brasília (UnB), Brasília, DF 70910-900, Brazil; rociocaceres05@hotmail.es (P.R.C.-V.); fascineli@yahoo.com.br (M.L.F.); razevedo@unb.br (R.B.A.); grisolia@unb.br (C.K.G.)

**Keywords:** nanotechnology, ecotoxicology, benthic species, metals, chronic toxicity

## Abstract

Silver nanoparticles (AgNPs) are used intensively in medical and industrial applications. Environmental concerns have arisen from the potential release of this material into aquatic ecosystems. The aims of this research were to evaluate the potential accumulation of silver in the whole body of organisms and analyze the effects of AgNPs on the survival and reproduction of the snail *Biomphalaria glabrata*. Results show slow acute toxicity with a 10-day LC_50_ of 18.57 mg/L and an effective decrease in the eggs and egg clutches per organism exposed to tested concentrations. Based on these data, the No Observed Effect Concentration (NOEC) observed was <1 mg/L for snail reproduction. For silver accumulation, we observed that uptake was faster than elimination, which was very slow and still incomplete 35 days after the end of the experiment. However, the observed accumulation was not connected with a concentration/response relationship, since the amount of silver was not equivalent to a higher reproductive effect. The data observed show that AgNPs are toxic to *B. glabrata*, and suggest that the snail has internal mechanisms to combat the presence of Ag in its body, ensuring survival and reduced reproduction and showing that the species seems to be a potential indicator for Ag presence in contaminated aquatic ecosystems.

## 1. Introduction

Silver is a rare element in the earth’s crust, which means that its presence is low. Thus, the release of a small mass of silver into water bodies by human activities can generate impacts on the natural conditions of the environment [1]. Different types of nanomaterials are currently incorporated into industrialized products, and among these are silver nanoparticles (AgNPs). They are widely used in the manufacture of textiles, materials to eliminate microorganisms and the odor from clothes, food packaging, water filters, paints, cosmetics, deodorants and biomedical products, mainly where antimicrobial properties are desirable [2,3,4]. Because of the need to continue using silver, thanks to its antimicrobial properties, the possible toxic effects of AgNPs against different organisms and non-target species should be investigated. Due to the classical accumulation of metals in living organisms, it is important to evaluate the potential bioaccumulation of Ag^+^, to help assess the predicted uses for this nanoscale material and to take measures in limiting activities that may present excessive risks to human health and the environment.

As observed in other studies with nanoparticles, the sedimentation and aggregation of part of the material occurs when it is in solution [5,6]. As affirmed by Klaine et al. [7], the ultimate sink for nanoparticles may be sediments in aquatic ecosystems and soils, and so there is a need to identify more model organisms that are appropriate for testing, among those that inhabit these ecosystems. Thus, aquatic snails, particularly *Biomphalaria* sp., are often used as test organisms, mainly because they move and graze on the bottom of water bodies, and because they have a benthic habit in the environment, which makes them ideal for testing contact with sedimentary materials [8].

The objective of this study, therefore, was to analyze the potential accumulation of silver in the whole body of this aquatic snail and the effects of AgNPs on its survival and reproductive performance.

## 2. Materials and Methods 

### 2.1. Characterization of Tested AgNPs

PVP-coated silver nanopowder 99.5% (CAS no “576832”) was purchased from Sigma-Aldrich, St. Louis, MO, USA. For morphology characterization, samples of silver nanoparticles (AgNP) were observed using transmission electron microscopy (TEM) (JEM-2100F, Tokyo, Japan). For this, the AgNPs were suspended in milliQ (MQ, Merck KGaA, Darmstadt, Germany) ultrapure water and sonicated for 40 minutes (Vibra-CellTM 75042, 20 kHz, 500 W). Subsequently, an aliquot of 25µL of the suspension was added to 975 µL of MQ water. Then, 4 μL of the dilution was placed in copper mesh (300 mesh) for analysis by transmission electronic microscopy (TEM). The AgNP diameters were obtained by analysis in the Motic Images Plus 2.0 computer program (Barcelona, Spain). The distribution of particle diameters was obtained using the best log-normal distribution adjustment.

### 2.2. Test Organisms

*Biomphalaria glabrata* (Say, 1818) is an aquatic snail that is native to Brazilian freshwaters, and it has been studied for a long time, because it is a Brazilian intermediate host of the parasite *Schistosoma mansoni* [9]. These snails are easily maintained in laboratory colonies, and several papers have proposed their use for ecotoxicity studies [10,11,12]. The organisms tested in the present research were acquired from the Laboratory of Ecotoxicology of Embrapa Cerrados in the Brazilian Federal District. 

### 2.3. Acute Toxicity Tests

To obtain the dilutions for the reproduction assay, an acute preliminary assay determined the lethal concentration for 50% of the organisms (LC_50_). Static acute assays lasting 96 hours were carried out in 3,000 mL beakers with reconstituted water (pH 7.3 ± 0.1, hardness 43 mg/L as CaCO_3_), maintained at 24 ± 1 °C under a 16-h light/8-h dark cycle and without food. Twenty adult snails were exposed (10 per beaker) to each concentration and mortality was evaluated.

### 2.4. Reproduction Assays and Silver Accumulation 

Assays took place with individually exposed adult snails (shell diameter 12 ± 3 mm), using ten organisms, in two replicates for each concentration, inside glasses of 300 mL, to test AgNPs at the concentrations of 0, 1.0, 2.5, and 5.0 mg/L in reconstituted water, with hardness of 40–43 mg/L as CaCO_3_. The exposure occurred in controlled environmental conditions at 24 ± 1 °C and dark/light cycle 8/16 hours, for four weeks. Test concentrations were renewed twice a week, and organisms were fed with lettuce leaves (approximately 1 cm^2^) plus 1 milligram of fish chow, available in fish shops, per organism. To count eggs and egg clutches, the glasses were internally coated with cellophane sheets, replaced on two days per week. The investigation involved recording lethality and the laying of eggs and egg clutches for 4 consecutive weeks. After this time, three snails were removed from each treatment, and the remaining snails stayed in reconstituted water without AgNPs until 15 and 35 days after the end of the experiment, for the quantification of silver in the snails’ body, as proposed by Oliveira-Filho et al. [13].

To investigate the accumulation of Ag, the content of Ag within the snail’s whole body was analyzed on the 15th day and on the 35th day after the end of the experiment. For this analysis, 3, 3 and 4 organisms, respectively, from each concentration, were extracted from the shell, washed in ultrapure water and stored at −20 °C, until subsequent analysis. Then, the samples from the groups were oven dried at 110 °C. For analysis of samples from each group, 0.5 g of dried samples were digested in 10 mL of concentrated nitric acid (HNO_3_, 65 % *v*/*v*), for 25 minutes at 200 °C. At the end of the digestion process, dilutions were performed using ultrapure water to obtain samples with a final volume of 10 mL. The Ag content was quantified by optical emission spectrometry with inductively coupled argon plasma (ICP-OES iCAP 7000 Series, Thermo Fisher Scientific, Waltham, MA, USA). To guarantee the quality of analysis, the analytical grade was maintained for all reagents and standards. Negative controls, duplicates, and analytical standards were used. The duplicate analysis for Ag showed concordance levels ranging from 77.8% to 98.1%. The recovery of metals from standards ranged from 96% to 105%, and the calibration coefficients were maintained at the ≥0.999 level.

### 2.5. Statistical Analysis

The lethal concentration for 50% of the organisms was calculated using the Trimmed Spearman Karber (Cincinatti, OH, USA, version 1.5) [14]. Variations between eggs and egg clutches produced by experimental groups were calculated by one-way ANOVA followed by the USEPA Dunnett Test (Cincinatti, OH, USA, version 1.5) [15,16] and Tukey test (SPSS Statistics, IBM, Armonk, NY, USA, version 20,). A t test was performed to determine differences in whole-body Ag between the control group and 15/35 days-after-exposure groups, and these differences were considered significant when *p* < 0.05.

## 3. Results and Discussion

### 3.1. Characterization of NPs

**Figure 1:**
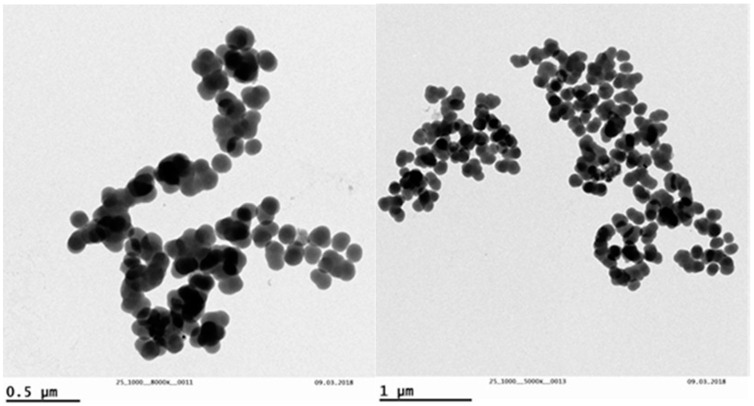
Characterization of the silver nanoparticles (AgNP) using transmission electron microscopy (TEM).

Aliquots from the same AgNPs were used by Cáceres-Velez et al. [17], and the present study obtained the same characterization results. TEM analyses allowed us to verify the shape and size of AgNPs (Figure 1). Nanoparticles of variable and irregular shape were observed, with a mean diameter of 115.17 ± 55.57 nm.

### 3.2. Acute Toxicity and Snail Reproduction Assays

Preliminary assays showed a relative delay in snails’ mortality. In the first 96 hours of exposure there was mortality only at the concentration of 100 mg/L. After that period, mortality continued to occur slowly, and the LC_50_ was obtained after 10 days. This aspect can be explained by the time of Ag^+^ release from AgNPs, as presented by Kittler et al. [18]. Then, the LC_50_ at 10 days was calculated at 18.57 mg/L (13.93–24.77), close to that obtained by Govindasamy and Rahuman [19] with the fish *Oreochromis mossambicus* after 8 days of exposure. On the other hand, Gonçalves et al. [20] found the LC_50_ at 96 hours among adult snails, *Physa acuta,* at a concentration of 7.05 mg/L. 

These authors tested AgNPs with diameters between 3 and 8 nm. In agreement with some review studies, the size and the shape of NPs are key factors in the toxicity [2,21,22]. In addition, *B. glabrata* snails tested in the present study are about double the size of the organisms tested by Gonçalves et al. [20], and all these aspects could explain the differences observed between the acute toxicity of AgNPs to *P. acuta* and *B. glabrata*.

After establishing that LC_50_ occurred at 10 days, the assays started for evaluation of the effects on reproduction and potential bioaccumulation. 

**Figure 2:**
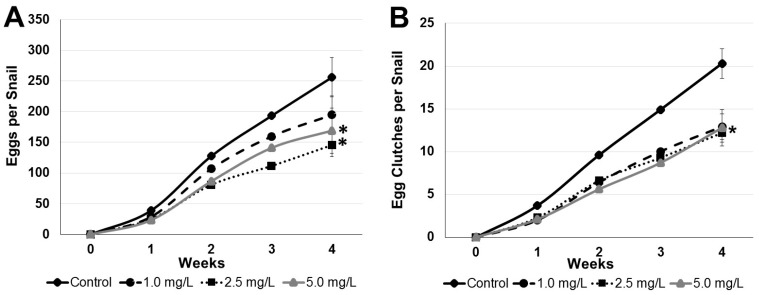
Effects of silver nanoparticles on the reproduction of *B. glabrata* snails (*n* = 10) per concentration. Data are presented as cumulative means of eggs (**A**) and egg-clutches (**B**) laid per snail (Mean ± SE). Differences (*p* < 0.05 ANOVA and Dunnett’s multiple comparisons test and Tukey test) between the control group and tested concentrations are indicated by an asterisk (*) at the fourth week.

The cumulative mean of eggs/snail (Mean ± SE) in the control at the end of 4 weeks was 253.6 ± 31.9, and at the concentrations of 1.0 mg/L (194.8 ± 30.4); 2.5 mg/L (145.9 ± 48.6); and at 5.0 mg/L (169.1 ± 30.3), respectively (Figure 2A). For egg-clutches/snail, the cumulative mean (Mean ± SE) in the control group was 20.3 ± 1.7, and at the concentrations of 1.0 mg/L (12.9 ± 1.5); 2.5 mg/L (12.6 ± 1.1); and at 5.0 mg/L (12.4 ± 2.1), respectively (Figure 2B).

After statistical determinations by Tukey test and Dunnett’s test, the results showed that there was significant inhibition in the number of eggs per individual at 2.5 mg/L in relation to the control at a level of *p* < 0.05 (Figure 2A). For egg-clutches per snail, there was significant inhibition at *p* < 0.05 in all concentrations (Figure 2B), suggesting a No Observed Effect Concentration (NOEC) < 1 mg/L for silver nanoparticles regarding the reproduction of *B. glabrata*.

In a similar study with the snail *Physa acuta* the authors observed low egg production in concentrations above 1 mg/L after 28 days of exposure, suggesting this concentration as the NOEC [23]. On the other hand, Gonçalves et al. [20] observed the effects on hatching success of the snail *P. acuta* and found an EC50 of 0.09 mg/L of AgNPs. Several previous studies have already indicated that hatching delay is the main and most susceptible end point in chronic bioassays using snails [8,11,12], although this effect did not arise in the present investigation.

### 3.3. Silver Accumulation

**Figure 3:**
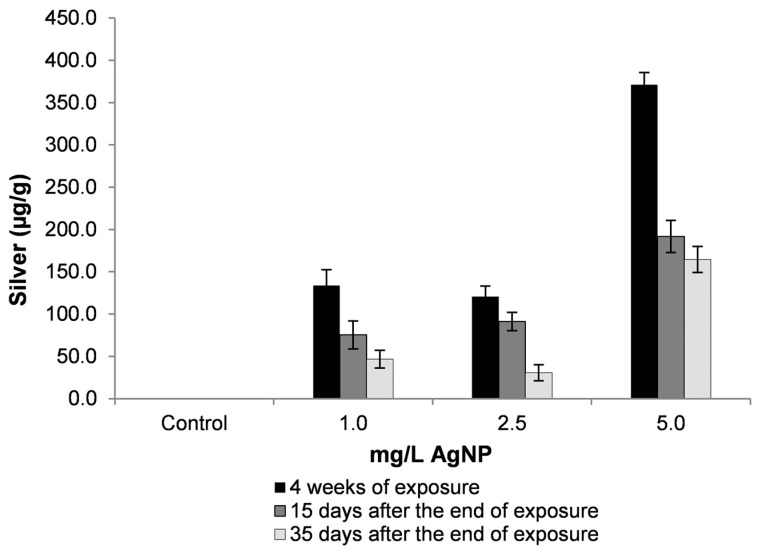
Amount of silver in snail’s body at different exposure times and concentrations regarding the tested AgNPs. Data are presented as means (±SD) in µg metal/g bodyweight.

Results observed in Figure 3 present the amount of silver in the whole body of snails after exposure to AgNPs. These data show how high the total amount of silver in the snail body can be in relation to the exposure concentration and to the control group. On the other hand, the elimination rate seems to be proportional, over time, after the experimental aquatic contamination had ended.

For mollusks, metal uptake is usually faster than release, and accumulation occurs in proportion to environmental concentrations [24]. As also demonstrated in Figure 3, although the amount of silver in the whole body of the snails exposed to 5 mg/L of silver nanoparticles is greater, this was not representative of a greater reproductive effect, since groups of lower concentrations showed the same inhibitory effect on the production of egg-clutches. 

With regard to toxicity, values of silver found in snails were higher than the lethal values of both AgNPs [20] and Ag ionic [25]. In a field study in contaminated sites in England, Langston and Zhou [26] observed values of 2.2 to 22.0 µg/g of Ag in marine gastropod snails, *Littorina littorea*. In this study, the authors did not report any adverse effects of metals on the snail.

Several studies have shown the modulation of AgNP toxicity by the sequestration of Ag by metallothionein in different species [27], including soil organisms [28,29], fish [30,31] and gastropod mollusks, such as the snail *Biomphalaria glabrata* [32]. In research with the estuarine polychaete *Nereis diversicolor*, Garcia-Alonso et al. [33] found that Ag from exposure to AgNPs was mainly associated with metallothionein. 

In the present study, the benthic habit of the tested organism leads to the supposition that the accumulation included not only the AgNP incorporated into the tissue, but also the AgNP retained in the gastrointestinal tract. However, in a study with the terrestrial snail, *Achatina fulica,* based on their modeling, Chen et al. [34] affirmed that dietary uptake is the dominant route for Ag accumulation in the case of AgNP exposure under most circumstances. Evaluating the accumulation of γ-Fe_2_O_3_ nanoparticles, Oliveira-Filho et al. [13] observed that the intestine of *B. glabrata* snails was full of the material after 30 days of exposure, without a significant effect on survival or reproduction. On the other hand, as indicated by Oliveira-Filho et al. [35] and Oliveira-Filho et al. [36], special attention should be given to possible predators of snails, which may have other forms of metabolism and effectively accumulate the metal.

Although studies have tried to understand the transformations of AgNPs in environmental compartments, it should be pointed out that biotransformation also occurs in the exposed species, mainly by digestive mechanisms in the gastrointestinal tract [37]. Braud et al. [38] describe the bacterium *Pseudomonas aeruginosa* as able to chelate silver, and Silva et al. [39] report the occurrence of this bacterium in the gastrointestinal microbiota of the snail *B. glabrata*.

Metal accumulation in the marine snail *Littorina littorea* showed that this aquatic gastropod is a good indicator of the non-essential elements Ag, Cd and Hg, reflecting industrial input. The peaks of these elements appeared in association with high molecular compounds, including enzymes and haemocyanin, and Ag and Hg were predominantly associated with insoluble material [26]. As pointed out by Wood et al. [40], speciation has a strong role in silver toxicity. In this research, the authors showed a 3000-fold decrease in the acute toxicity of Ag^+^ to rainbow trout after it had been complexed by thiosulfate.

As seen in the present study, AgNPs in higher concentrations were lethal only after 10 days of *B. glabrata* exposure (LC_50_ = 18.57 mg/L), and this can be explained by the slow time release of Ag^+^ from AgNPs [18]. However, in lower concentrations after an exposure of 30 days (NOEC < 1.0 mg/L), AgNPs inhibit the reproduction of the snails, and the organisms accumulate Ag^+^ in concentrations higher than that tested, indicating that the freshwater snail has some metabolic mechanism that mitigates silver acute toxicity.

## 4. Conclusions 

To summarize, the results show that silver nanoparticles present adverse effects on survival, and at non-lethal concentrations, they can affect the reproduction rate of the aquatic snail *B. glabrata*. In addition, it was seen that this species accumulates silver, but it is capable of slowly releasing the metal in clean water. Overall, the data obtained indicate that this benthic aquatic grazer presents internal mechanisms against silver acute toxicity that need more investigation, especially regarding the transformation processes of AgNPs. However, the present study suggests that the species tested could be a potential indicator for the detection of silver in a contaminated environment.
